# The influence of the COVID‐19 pandemic on the demand for different shades of green

**DOI:** 10.1002/pan3.10304

**Published:** 2022-02-14

**Authors:** Kathleen K. L. Yap, Malcolm C. K. Soh, Angelia Sia, Wei Jun Chin, Sophianne Araib, Wei Ping Ang, Puay Yok Tan, Kenneth B. H. Er

**Affiliations:** ^1^ National Parks Board Singapore Botanical Gardens Singapore City Singapore

**Keywords:** COVID‐19, cultural ecosystem services, nature preferences, park usage patterns, urban green space

## Abstract

1. COVID‐19 has heightened the dependence of urban dwellers on cultural ecosystem services provided by urban green spaces (UGS), specifically in regard to the provision of recreational opportunities, and psychological and physical health benefits arising from their use.
2. As different types and levels of cultural ecosystem services are provided by different types of UGS, people may seek out different UGS to satisfy personal needs over various phases of COVID‐19 mobility restrictions imposed by cities. We report on a study that took advantage of the different phases of COVID‐19 mobility restrictions to assess the demand for and perception of different types of UGS in Singapore.
3. The study utilised four datasets to compare demand for and visitorship patterns of UGS before the pandemic (Pre‐Circuit Breaker), the duration of the strictest mobility restrictions (Circuit Breaker), and after the measures were relaxed (Post‐Circuit Breaker). We used Google Search trends as a proxy for UGS demand, Google mobility data for an overview of population visitorship trends, visitor counts for granular insights on actual visitorship trends, and qualitative data on perception of parks by park visitors after restrictions eased. Parks were categorised as manicured and less manicured UGS for analysis.
4. Search interest for UGS overall fell by more than 50% from during Circuit Breaker but the post‐Circuit Breaker levels exceeded pre‐Circuit Breaker, with a 70.9% increase for less manicured UGS compared to 20.8% for manicured UGS. This corroborated with Google mobility and visitor counts, which showed a steep decrease in park use followed by a rapid increase in the same periods, and with increased visitorship in the less manicured UGS. The perception study also showed that more than 50% of respondents reported visiting parks that they have never visited before, and there was a greater appreciation and use of UGS after the pandemic and preference for less manicured and more naturalistic landscapes.
5. The pandemic has heightened the demand for cultural ecosystem services provided by UGS. Our study showed that this demand is not uniform across different types of UGS, with an increase visitorship and preference for less manicured green spaces.

Read the free Plain Language Summary for this article on the Journal blog.

## Abstract 2

Read the free Plain Language Summary for this article on the Journal blog.

## 1 INTRODUCTION

The emergence of COVID‐19 has impacted the world on an unprecedented scale. The fear of infection, coupled with disruptive lockdown measures introduced by governments worldwide, has disrupted the livelihood and lifestyle of urban populations and led to increasing reports on the negative psychological impacts of COVID‐19 on urban dwellers (Passavanti et al., 2021; Xiong et al., 2020). These impacts often manifest as higher than normal rates of anxiety, depression and post‐traumatic stress disorder among the general population (Giuntella et al., 2021). This has led the American Psychological Association to describe COVID‐19 as an epidemiological and psychological crisis, with attendant calls for appropriate government responses (American Psychological Association, 2020).

Against this backdrop, there is also heightened awareness of the role of urban nature in mitigating the impacts of COVID‐19 on health and well‐being. Studies globally suggest that the pandemic has sparked an increased demand for urban green spaces (UGS). Kleinschroth and Kowarik (2020) observed a sharp spike in interest for outdoor recreation after travel restrictions were imposed, with proportionally higher Google search trends for outdoor‐related activities (e.g. hiking), compared to other leisure activities (e.g. shopping). People's desire to visit UGS did not diminish in areas with restricted public access to parks (Zhu & Xu, 2020). Even in cities where UGS was not readily accessible, people were willing to travel longer distances to utilise green spaces outside their immediate living environment (Ugolini et al., 2020). Increases in visits were also accompanied by reports of the perceived importance of UGS (Geng et al., 2020), especially as an avenue to reduce stress (Grima et al., 2020) and strengthen resilience and support mental health (Aerts et al., 2021).

Such widespread observations of a sudden shift in social behaviours following onset of the pandemic highlight the importance of the relationship between people and their environment, in particular, that of urban nature, which is mediated by cultural ecosystem services (CES). While the various tangible benefits of UGS have been well documented under the umbrella of urban ecosystem services (Gómez‐Baggethun et al., 2013), CES are relatively less understood, but yet recognised to be equally integral to pursuits of urban sustainability and liveability (Andersson et al., 2015). CES, as the intangible and non‐material benefits provided by nature, are theoretically more challenging to quantify (Fish et al., 2016) as it is highly dependent on the cultural background of distinct communities, as well as the environment (Hirons et al., 2016; Plieninger et al., 2013).

The studies cited above (Geng et al., 2020; Grima et al., 2020; Ugolini et al., 2020) provided significant evidence that CES has become more valuable to people during the pandemic. This is reflected in the increase visitorship to UGS and perception of the importance and benefits of UGS (Ma et al., 2021; Venter et al., 2020). While UGS have been shown to be valuable in most socio‐cultural context, these studies do not explore whether different types of UGS meet different needs of urban dwellers in a unique situation like the pandemic. We should expect that the type of UGS would confer a different extent of effect to its users and reciprocate varying responses. For example, the size of forest in urban areas affects the relationship between green space and mental health (Akpinar et al., 2016). Thus, do people seek out different types of green spaces during the pandemic, and how might such an understanding inform the management and planning of UGS for cities?

We used the opportunity presented by the imposition of different levels of mobility restrictions in Singapore during the pandemic to address these questions by undertaking a study that combined different data sources. Singapore is an interesting case for such a study for various reasons. First, it is widely regarded as a well‐planned and green city (Tan et al., 2013), with a high level of park usage (Petrunoff et al., 2021), and a recent report suggests that Singapore residents do not have an extinction of experience with nature (Oh et al., 2020). In other words, Singapore residents are generally not deprived of contacts with nature. Given the already high baseline level of park use and exposure to nature, it is interesting to examine whether COVID‐10 mobility restrictions have affected park visitorship and preference. Second, as a high‐density, compact tropical city, a study on Singapore contrasts with the large majority of studies on UGS uses during COVID‐19 that have been undertaken in temperate climates. As geography, in addition to socio‐economic context, is known to influence CES from UGS (Ho et al., 2005; Ono et al., 2021), results of studies in temperate regions may not be transferrable to Singapore. For instance, we note that the increase in park use after the onset of COVID in temperate countries (after January 2020) coincided with warmer temperatures and can possibly be a confounding factor in understanding UGS visitorship patterns and demands during COVID‐19 (Rice & Pan, 2021). A study on Singapore can provide insights on differences in green spaces usage and preferences across socio‐economic, cultural and climatic contexts.

The primary objective of the study is to assess the relative demand for and visitorship pattern of Singapore to manicured and less manicured parks over different phases of mobility restriction, with the aim of developing insights on how different types of urban green spaces may satisfy different needs of the population. We define manicured parks as parks that have most of its area covered with frequently maintained greenery and built park amenities. Conversely, less manicured parks refer to parks that have most of its area covered with greenery that is not maintained, that is, with higher quantum of natural habitats and spontaneous vegetation, and more naturalistic looking landscapes. We first examined the impact of the COVID‐19 restrictions in Singapore on the travel mobility using data provided by Google community mobility data. We then assessed the interest levels of UGS through the use of Google search trends and compared this with actual visitorship to selected parks before and during the pandemic. To obtain a qualitative understanding of the trends, we conducted a survey of park visitors to assess their motivations to visit UGS during the pandemic. The study showed that 63.2% of respondents reported having visited parks that they have never visited before, and ‘exercise’, ‘appreciate landscape’, ‘spend time with others’ and ‘watch wildlife’ being the most cited reasons for visiting parks.

## 2 METHODS

### 2.1 COVID‐19 mobility restrictions in Singapore for parks and outdoor spaces

Like many other cities, Singapore imposed strict restrictions from 7 April 20 to 1 June 20, termed the ‘Circuit Breaker’, to control the outbreak of COVID‐19. During this period, other than essential services, citizens were mandated to work from home and social activities severely curtailed with the closure of retail malls and shops, restaurants, sports recreation spaces and attractions. Citizens could only leave their homes for essential activities such as to seek medical attention, get food and exercise. These restrictions remained until 18 June 2020. Although parks remained open during the Circuit Breaker, there were several restrictions. For example, exercise in groups was disallowed—people could only use parks to exercise alone or with one other household member. Recreational activities, such as picnicking or ball games, were prohibited. Open spaces and recreational facilities such as lawns, beaches, and playgrounds were closed to deter social gatherings. Carpark facilities in parks were also closed to encourage visitors to choose nearby parks over those that were further. Visitorship to parks was monitored daily, and entry was denied if crowd levels were observed to be high.

### 2.2 Online interest in parks

To identify whether the pandemic had any influence on interest in parks in Singapore, we analysed Google search trends between January to September 2020 to observe shifts in interest for specific parks. Google Trends have been used by studies as a proxy for market interest to predict demand, even for visits to attractions (Höpken et al., 2019). Google search trend is a useful indicator to predict consumer trends as it informs how the proportional number of searches changes over time. Here, we used it to monitor and compare the effect of the mobility restrictions on interest in parks across Singapore.

In all, 44 parks that were identified to serve the population beyond the immediate neighbourhood were selected and their names keyed into the Google Search web interface (http://trends.google.com) to obtain search trend data between 5 January and 3 October 2020. All searches for park names were keyed in as a ‘topic’ query and not a ‘search term’ query, as the former retrieves searches that refer to the same topic, in this case a location, whereas the latter retrieves related searches with similar words (Google Trends, n.d.). After searching, we checked the top five related queries for each park name to ensure that the queries are referring to the park and not referencing other locations with similar names. Then the trend data of 9 months were downloaded as a 7‐day value. Of the 44 parks, 26 parks were removed from the analysis of which three parks were not recognised as ‘topics’ and 23 parks returned with insufficient data to show related queries, due to a low volume of search results. As the Google search trends provide values based on proportion on a scale of 100, all the searches were standardised on the same scale using the ‘compare’ function.

Parks were categorised into two broad classifications of manicured and less manicured park based on their predominant type of landscape (Table 1). Manicured parks are defined as green spaces that are dominated by frequently maintained ornamental green spaces, with natural habitats and/or spontaneous vegetation occupying less than 50% of the total park area. Less manicured parks are defined as parks where more than 50% of the total park area comprises of natural habitats and/or spontaneous vegetation, with hiking or walking trails. These habitats may include tropical rainforest, secondary forests and woodlands, mangrove, mudflats, grasslands and coastal vegetation. These parks are all types of UGS as they are situated within the urban landscape matrix in Singapore.

**TABLE 1:** Description of analysed parks. Abbreviations of park names in parentheses. Study method refers to the data used for each park where GS = Google trend, VS = visitor count, Q = questionnaire

Park name	Study method	Size (ha)	Category	Broad description
Bukit Timah Nature Reserve (BTNR)	GT, VC, Q	163	Less manicured	Primary and secondary rainforest on hilly terrain
Sungei Buloh Wetland Reserve (SBWR)	GT	130	Less manicured	Mangrove, mudflats, ponds and forests
Mount Faber Park (MFP)	GT	56.5	Less manicured	Secondary rainforest on a hilly terrain
Coney Island (CI)	GT	50	Less manicured	Coastal forests, woodlands, grasslands and mangroves
Labrador Nature Reserve (LNR)	GT	22	Less manicured	A secondary rainforest with rocky shore habitat
Admiralty Park (AP)	GT	27	Less manicured	Secondary forest, grasslands and mangroves
Kent Ridge Park (KRP)	GT	46.5	Less manicured	Secondary rainforest on a hilly terrain
Windsor Nature Park (WNP)	Q	75	Less manicured	Secondary rainforest
Fort Canning Park (FNP)	GT	17.9	Manicured	Historical park with mature greenery
Singapore Botanic Gardens (SBG)	GT, VC, Q	63	Manicured	Garden, with botanical collections and expanse of lawns
Jurong Lake Gardens (JLG)	VC, Q	53	Manicured	Garden that features naturalistic landscapes, playgrounds and expanse of lawns
East Coast Park (ECP)	GT	186	Manicured	Large expanse of beach interspersed by lawns and recreational facilities, with some patches of spontaneous vegetation
Bishan‐Ang Mo Kio Park (BAMK)	GT, Q	62	Manicured	Mostly manicured green spaces, with naturalistic riverine landscape
Hort Park (HP)	GT	2.3	Manicured	Mostly manicured green spaces, with horticultural displays
West Coast Park (WCP)	GT	50	Manicured	Mostly manicured green spaces, with beach, small patch of mangroves
Punggol Waterway Park (PWP)	GT	12.3	Manicured	Mostly manicured green spaces with recreational facilities
Pasir Ris Town Park (PRTP)	GT	14	Manicured	Mostly manicured green spaces with recreational facilities
Changi Beach Park (CBP)	GT	31.1	Manicured	Large expanse of beach, interspersed with lawns and recreational spaces
Punggol Park (PP)	GT	16.3	Manicured	Mostly manicured green spaces with recreational facilities
Woodlands Waterfront Park (WWP)	GT	11	Manicured	Mostly manicured green spaces with recreational facilities

### 2.3 Population visitorship trends

To assess the trend in visitorship to parks, we obtained mobility data from Google that is publicly available for the months of February to October 2020, for the purpose of understanding potential risks of COVID‐19 spread in communities (Google LLC, n.d.). The data for Singapore were retrieved from Our World in Data (n.d.). Google's community mobility data provides anonymised general population visitorship trends to various categories of locations, with parks being one of the categories. The anonymised information is obtained from smartphone users who have permitted Google to use GPS tracking on their phones, and counts a visit to a location when the person is within the geographical area. The overall mobile subscription rate in Singapore stands at 152% of the overall population, as of September 2020 (IMDA, n.d.). Hence, our data would be an accurate representation of visitorship trends. The data were presented as a change in visitorship percentage compared to the pre‐COVID‐19 baseline, which was from 5 January to 3 February 2020.

To complement the Google mobility trend data, we also obtained visitor counts from selected parks, from 5 January to 26 September 2020. The visitor counts for each park were obtained from video surveillance cameras (Avigilon, model no. 2.0C‐H4SL‐B01‐IR) at major park entrances that monitor visitor entry 24 hr a day and counted using in‐built video analytics (software ICONICS GENESIS64). Three parks had visitorship data before the Circuit Breaker, namely Bukit Timah Nature Reserve (BTNR), Singapore Botanic Gardens (SBG) and Jurong Lake Gardens (JLG). These parks are popular green spaces and have high recreational value. They are similar in the extent of accessibility—each within short walking or cycling distances, or easily accessible by bus and train (stations are <0.85 km from park entrance). Since the beginning of the outbreak, 90 parks have deployed counting systems to monitor crowd levels to support safe distancing measures. However, these parks were not included in this study as there was no comparative visitor counts before the Circuit Breaker.

### 2.4 Onsite survey of park visitors

To understand the motivations for visiting parks during the Circuit Breaker and post‐Circuit Breaker period, we carried out on‐site surveys at five parks that vary in the degree of naturalness (i.e. from less manicured to more manicured). These were conducted at the BTNR, SBG, JLG, Windsor Nature Park (WNP) and Bishan‐Ang Mo Kio Park (BAMK). Surveys were conducted over 6 weeks during the post‐Circuit Breaker period between August and September 2020, on one weekday and one weekend (Saturday or Sunday) each week. Surveys were carried out in the mornings (8 am–12 pm) and evenings (3 pm–7 pm), to cover a wider range of visitors. The survey was given at the common spaces in each park, such as entrances of the park and outside public restrooms, to ensure that the study sample is not biased towards any particular type of user.

The survey questions (Table 2) included self‐reported park visitor patterns, motivations for visit and perceptions on park usage since the Circuit Breaker. A binary (Yes/No) answer choice was used for perception questions instead of a Likert scale to reduce the likelihood of respondents inserting personal nuances to the answers. Park staff were given a script to explain the purpose and delivery of the anonymous survey, seek verbal consent for voluntary participation and assure participants that all responses are non‐identifiable. After verbal consent was received, the participants were provided via a QR code to scan on their personal devices that led to the online survey (this was done to ensure safe distancing and added confidentiality of responses). The online form had a cover letter that reiterated that the survey is anonymous and participation is voluntary. They were also informed that did not have to submit the form should they feel uncomfortable with any questions. By competing the survey, respondents consented to NParks collecting and using the information for the study. Only verbal consent to proceed was obtained. No incentives were given to respondents for completing the survey. The survey was hosted on a government online platform and all responses were encrypted end‐to‐end and accessible only by the research team. There was no local research ethics committee available to review this survey. However, the survey was done in a public park and does not put respondents at more than the minimal risks of everyday of life.

**TABLE 2:** Questions asked in on‐site survey

Multiple choice questions
What mode of transportation do you usually use to travel to this park?
How much time does it take you to travel to this park to using the above‐mentioned transportation mode?
How often do you visit this park since the start of Circuit breaker (on average)?
How has the frequency of your visit to this park changed, comparing now and before the Circuit Breaker?
What do you enjoy doing at this park?
Which of the following are reasons that motivate you to visit the parks during and after the Circuit Breaker?
Yes\no questions
I resided in Singapore before and during the COVID pandemic Since April, I visited parks that I never visited before, or parks that I have not been to in the last 1 year
I appreciate parks and the greenery in my neighbourhood now more than before the pandemic
I am happy seeing our roadside and open spaces grow a little wilder
I will continue to visit parks just as often or more even when things become normal again
I feel that this park has become more crowded than before Circuit Breaker
I am aware of some of the measures in parks to maintain public safety during the pandemic
I feel that the measures in parks to ensure public safety during the pandemic are sufficient

### 2.5 Data preparation

Singapore is a tropical city‐state 1° north of the equator, with relatively consistent weather throughout the year. Hence, we assumed that any significant changes to park visitorship were likely due to social responses to COVID‐related policies rather than weather fluctuations. The mean rainfall per month during the sampling months (January to October) range from 105.1 to 221.6 mm, while the temperature ranges from 24.3 to 32.4°C (Meteorological Service Singapore, n.d.).

Data from 5 January and 3 October 2020 were standardised to weekly counts to account for variation across weekdays and weekends. To discern the effects of Circuit Breaker, time periods were categorised into three specific windows by week relative to the Circuit Breaker: Pre‐Circuit breaker (Weeks 1–13, 7 January to 6 April 2020), Circuit Breaker (Weeks 14–23, 7 April to 15 June 2020), and post‐Circuit Breaker (Weeks 24–39, 16 June to 3 October 2020). Within these periods, there were a similar number of public holidays with 2 days in Pre‐Circuit Breaker and 3 days each in Circuit Breaker and Post‐Circuit Breaker. A summary of the data used and the respective dates of data collection is in Table 3.

**TABLE 3:** Summary of the data used and dates of data collection which include Google trend searches (GT) from 5 January to 3 October 2020, Google mobility data (GM) from 17 February to 3 October 2020, visitor counts (VC) from 5 January to 26 September 2020, and onsite questionnaires (Q) from 26 august to 4 October 2020

Pre‐circuit breaker (5 January–6 April 2020)	Circuit breaker (7 April–18 June 2020)	Post‐circuit breaker (19 June–4 October 2020)
GT, GM, VC	GT, GM, VC	GT, GM, VC, Q

### 2.6 Statistical analysis

Two‐sample unequal variance Student's *t*‐test was used to compare the difference between pre‐Circuit Breaker and post‐Circuit Breaker Google search proportions for parks.

Beta generalised linear mixed model (GLMM) with logit link function was then fitted to determine the effects of Circuit Breaker on the Google search proportions (Bolker et al., 2009). The number of shops open, size of park, park type and Circuit Break phases (i.e. pre‐Circuit Breaker, Circuit Breaker and post‐Circuit Breaker) were included in our model as fixed effects, while park name was designated as a random effect. The number of shops open refers to the number of food and beverage and retail outlets in the park that were operating in our study period, which may have affected the search trends for parks with such amenities.

Chi‐square test was used to determine whether there were significant differences between the less manicured and manicured park types across the three Circuit Breaker phases for Google search proportions data and park visitor counts. Chi‐square post‐hoc test with Bonferroni correction was used to determine which Circuit Breaker phase had significant proportional differences (Beasley & Schumacker, 1995). Chi‐square test and the chi‐square post‐hoc test were also applied to compare the results of the questionnaire surveys.

Two‐sample unequal variance Student's *t*‐test, GLMM and Chi‐square analyses were conducted in R (v 4.0.2) (R Core Team, 2020). We used the ‘mass’ package (v 7.3‐53) for our Chi‐square test (Ripley et al., 2020), ‘chisq.posthoc.test’ package (v 0.1.3) to perform our post‐hoc Chi‐square tests (Ebbert, 2019), and the ‘glmmTMB’ package for our GLMM mixed‐effects model (v 1.0.2.1) (Magnusson et al., 2020).

## 3 RESULTS

### 3.1 Effect of circuit breaker on interest in parks

The proportions of Google searches for parks varied across the 18 individual parks (Table 4). Before the Circuit Breaker, the top three most searched parks were all manicured parks. The mean proportional search for the manicured parks was also higher than the less manicured parks by 41.3%. During the Circuit Breaker, searches for parks generally decreased by half, across less manicured and manicured parks. After the Circuit Breaker, there was a surge in interest for parks as Google searches surpassed the pre‐Circuit Breaker search proportions for all parks, except SBG. The increase in searches varied greatly across the parks, from 9.3% to as high as 121.2%. Both BTNR and SBWR, which are nature parks and hence less manicured, became the top second and third most searched parks post‐Circuit Breaker. They were ranked 4th and 7th pre‐Circuit Breakers, respectively.

**TABLE 4:** Average Google Trends search proportions for less manicured (*n* = 7) and manicured parks (*n* = 11) during the different circuit breaker phases, ranked from the highest to lowest search proportions for each park type. Percent changes in the search proportion compared to pre‐circuit breaker are shown in parentheses. *** indicates significant differences between circuit breaker/post‐circuit breaker and pre‐circuit breaker at *p* < 0.001; *p < 0.05

Park names	Pre‐circuit breaker	Circuit breaker	Post‐circuit breaker
Manicured parks			
ECP	49.46	17.55 (−64.52%)***	66.06 (+33.56%)***
SBG	25.23	8.40 (−66.71%)***	18.13 (−28.14%)***
FCP	21.08	8.50 (−59.68%)***	23.04 (+9.28%)
BAMK	9.46	6.80 (−28.12%)*	15.94 (+68.50%)***
PWP	9.15	3.80 (−58.47%)***	10.38 (+13.44%)
HP	9.08	3.40 (−62.56%)***	11.06 (+21.81%)
PRTP	8.69	4.20 (−51.67%)***	10.25 (+17.95%)
WCP	8.62	3.30 (−61.72%)***	10.69 (+24.01%)
CBP	4.31	1.80 (−58.24%)***	6.44 (+49.42%)*
PP	3.85	2.60 (−32.47%)***	6.38 (+65.71%)***
WWP	2.77	1.50 (−45.85%)	4.88 (+76.17%)***
Mean of all manicured parks	13.79	5.62 (−59.23%)***	16.66 (+20.79%)
Less manicured parks			
BTNR	15.23	9.10 (−40.25%)***	25.44 (+67.04%)***
MFP	14.15	5.00 (−64.66%)***	21.06 (+48.83%)***
LNR	11.77	3.50 (−70.26%)***	14.06 (+19.46%)
SBWR	10.85	4.20 (−61.29%)***	24.00 (+121.20%)***
CI	9.54	5.00 (−47.59%)***	21.00 (+120.13%)***
AP	4.00	1.70 (−57.50%)***	6.06 (+51.50%)*
KRP	2.77	2.60 (−6.14%)	5.13 (+85.20%)***
Mean of all less manicured parks	9.76	4.44 (−54.47%)***	16.68 (+70.91%)***

Although the mean search proportions for less manicured and manicured parks were fairly equal in post‐Circuit Breaker, only the less manicured parks had a significant increase of 70.9%, compared to pre‐Circuit Breaker (*t* = −6.21, *df* = 174.29, *p* < 0.001). Manicured parks saw a much lower Google search increase of 20.8% in post‐Circuit Breaker, compared to pre‐Circuit Breaker (*t* = −1.63, *df* = 316.97, *p* = 0.10). Google searches for both less manicured and manicured parks were significantly affected during Circuit Breaker (less manicured parks, *t* = 7.94, *df* = 147.98, *p* < 0.001; manicured parks, *t* = 6.34, *df* = 197.16, *p* < 0.001).

**FIGURE 1:**
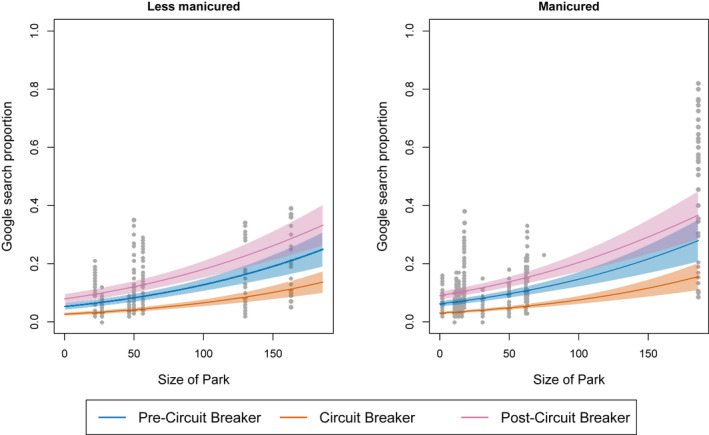
Effect of the size of the park and circuit breaker phases on Google search proportions as predicted by size of park and change in circuit breaker phases using beta GLMM. The standard errors and observed values are represented as shaded polygons and grey dots, respectively

Model comparisons revealed that the most parsimonious model included all factors except park type (AIC_c_ = −2,757.2, weight = 0.672). The next best model included all factors (AIC_c_ = −2,755.8, weight = 0.328). All factors except for park type showed a significant relationship with the Google Trends search proportions (Table 5). Circuit Breaker had the largest effect followed by the post‐Circuit Breaker period (Table 5). The GLMM plots showed that the effect of park size, as well as the Circuit Breaker and post‐Circuit Breaker phases, influenced the Google search trends for both less manicured and manicured parks (Figure 1). Both the number of shops that were open and the size of the park also had significant effects on the searches, with a positive correlation (Figure 1). Overall, manicured parks had a higher search proportion compared to less manicured parks, although this was not statistically significant (Table 5).

**TABLE 5:** Coefficient estimates, standard error (*SE*) and *z* values for fixed effects covariates of beta GLMM with Google search proportions as our response variable. Categorical covariates which include circuit breaker and post‐circuit breaker are compared with pre‐circuit breaker while manicured parks are compared with less manicured parks. *** indicates significant effects at *p* < 0.001

	Coefficient estimate	*SE*	*z* value
Intercept	−2.93	0.25	−11.60
Circuit Breaker***	−0.75	0.12	−6.32
Post‐Circuit Breaker***	0.40	0.10	4.07
Manicured Park (park type)	0.15	0.24	0.63
No. of Shops open***	0.04	0.005	7.83
Size of park (ha)***	0.01	0.002	4.19

### 3.2 Effect of circuit breaker on actual park visitor patterns

**FIGURE 2:**
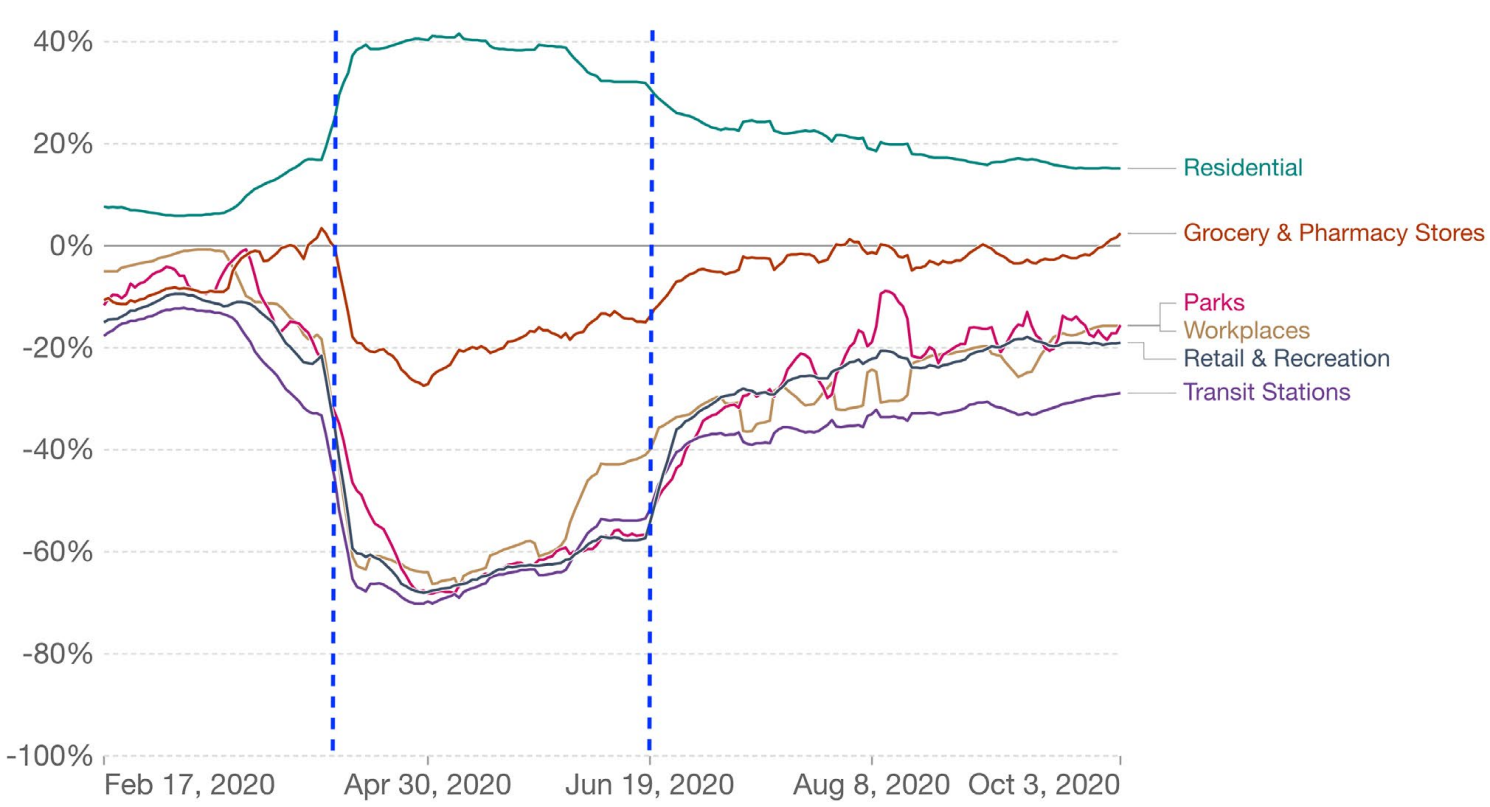
Reported visitor change to parks and other categories of places measured by Google community mobility data (Google LLC) and chart extracted from our world in data website compared to the median visitor level between 3 January and 6 February 2020. The blue vertical dotted lines represent the start of circuit breaker (7 April 2020) and subsequently phase 2 (19 June 2020)

The Google Mobility Data reflected a steep decline in visitors to all areas of interest outside of residence upon implementation of the Circuit Breaker on 7 April 20, with the exception of grocery and pharmacy stores (Figure 2). Residents in Singapore were generally compliant in adhering to the advisories and staying home in general, which resulted in the synchronised drop in outdoor visits and activities all around (Yip et al., 2021). Visitors to parks decreased more gradually than other categories of places, and hit the lowest point on 1 May 20, with a decrease of 68% from the baseline value taken between 3 January and 6 February 20. Similarly, the easing of regulations on 19 June 20 saw a sudden increase in visitors during the first 2 weeks, followed by a gradual increase. A month after the Circuit Breaker, the increase in visitors to parks was in pace and slightly higher than the increases for workplaces, retail & recreation, in contrast to the pre‐Circuit Breaker trends.

Although the Google Mobility Data reflect that the visitors to parks exceeded the baseline rate taken between 3 January and 6 February, the baseline would have been affected by tourism, which took a sharp hit due to the travel bans globally. Tourist numbers in Singapore were 1.69 million visitors the month of January 2020, and dropped to 8,912 visitors in the month of August 2020 (Elangovan, 2020).

Visitor counts from JLG, SBG and BTNR showed a slightly different visitor trend from the Google mobility data. On Google's website, examples of locations that are considered in the park category include local parks, national parks, public beaches, marinas, dog parks, plazas and public gardens. These are not clearly defined, and it is possible that Google data encompassed a broader scope of locations that include parks which had major tourist attractions that remained closed during this time (e.g. Gardens by the Bay).

**FIGURE 3:**
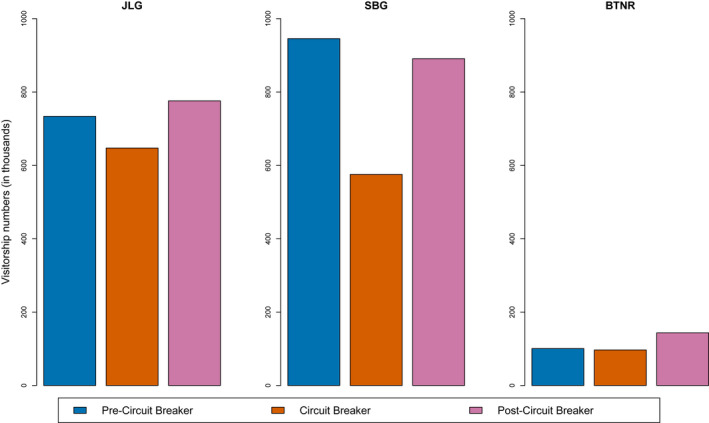
Relative visitor counts to three well‐known parks that remained open throughout the pandemic were significantly different between pre, during and post‐circuit breaker. Chi‐square post‐hoc tests indicate the differences between phases and parks were significant (*p* < 0.001 for all except Singapore botanic gardens where *p* < 0.05)

The visitor count trend across the three parks were also significantly different (*χ* ^2^ = 32,426, *df* = 4, *p* = 0). During the Circuit Breaker, SBG showed a visible drop in the visitors, while BTNR and JLG maintained visitor levels at about the same as pre‐Circuit Breaker. Post‐Circuit Breaker, BTNR saw a surge in visitor numbers, 42.6% increase compared to pre‐Circuit Breaker numbers. On the other hand, JLG saw a moderate 5.8% increase in visitor numbers, while SBG experienced a decline, compared to pre‐Circuit Breaker numbers (Figure 3).

### 3.3 Park visitorship patterns

A total of 1,196 respondents were interviewed for the questionnaire survey in five parks. Of these, 45 respondents said they did not live in Singapore during or before the Circuit breaker and were excluded, leaving a total of *n* = 1,151. Respondents were 15 years old and above. Most of the respondents were between 20 and 59 years old (80.9%), 7.2% of respondents were aged 15–19 years old and 11.9% were 60 years old and above. 86.5% were Singapore Citizens or Permanent Residents and the remaining were foreign pass or permit holders.

When asked whether the frequency of their visit to the park had changed compared between post‐Circuit Breaker and pre‐Circuit Breaker, close to 45.4% responded that there was no difference, while 33.4% responded that they visited the park more frequently than before (*χ* ^2^ = 7.40, *df* = 2, *p* = 0.025; Table 6). 97.9% of all respondents felt that they will continue to visit parks just as often, or more in the new normal (*χ* ^2^ = 686.1, *df* = 1, *p* < 0.001). Most respondents (63.2%) also reported that since the Circuit Breaker, they have visited parks that they have never visited before or in the last one year (*χ* ^2^ = 41.15, *df* = 1, *p* < 0.001; Table 6).

**TABLE 6:** Park use patterns and perceptions of park visitors (*n* = 1,151). Significant differences from the null hypothesis where proportions were equivalent across the responses are highlighted; *** indicates significant differences at *p* < 0.001; *p < 0.05

	Responses					*p*‐value
Park use						
Activity	Exercise	Appreciate Landscape	Spend time with others	Watch wildlife	Others	
	836	723	569	372	46	***
Motivation	To relax	Bored at home	Malls were closed	Others		
	971	331	37	111		***
Frequency	>1–2 times a week	Once a week	1–2 times a month	First time		
	248	207	263	433		***
Change in frequency	More frequently	No difference	Less frequently			
	384	523	244			*
New parks		Yes	No			
		728	423			***
Perception		Yes	No			
Greater appreciation of UGS		1,050	101			***
Wild roadside greenery		1,085	66			***
Continue or increase frequency of visit in new normal		1,127	24			***

**FIGURE 4:**
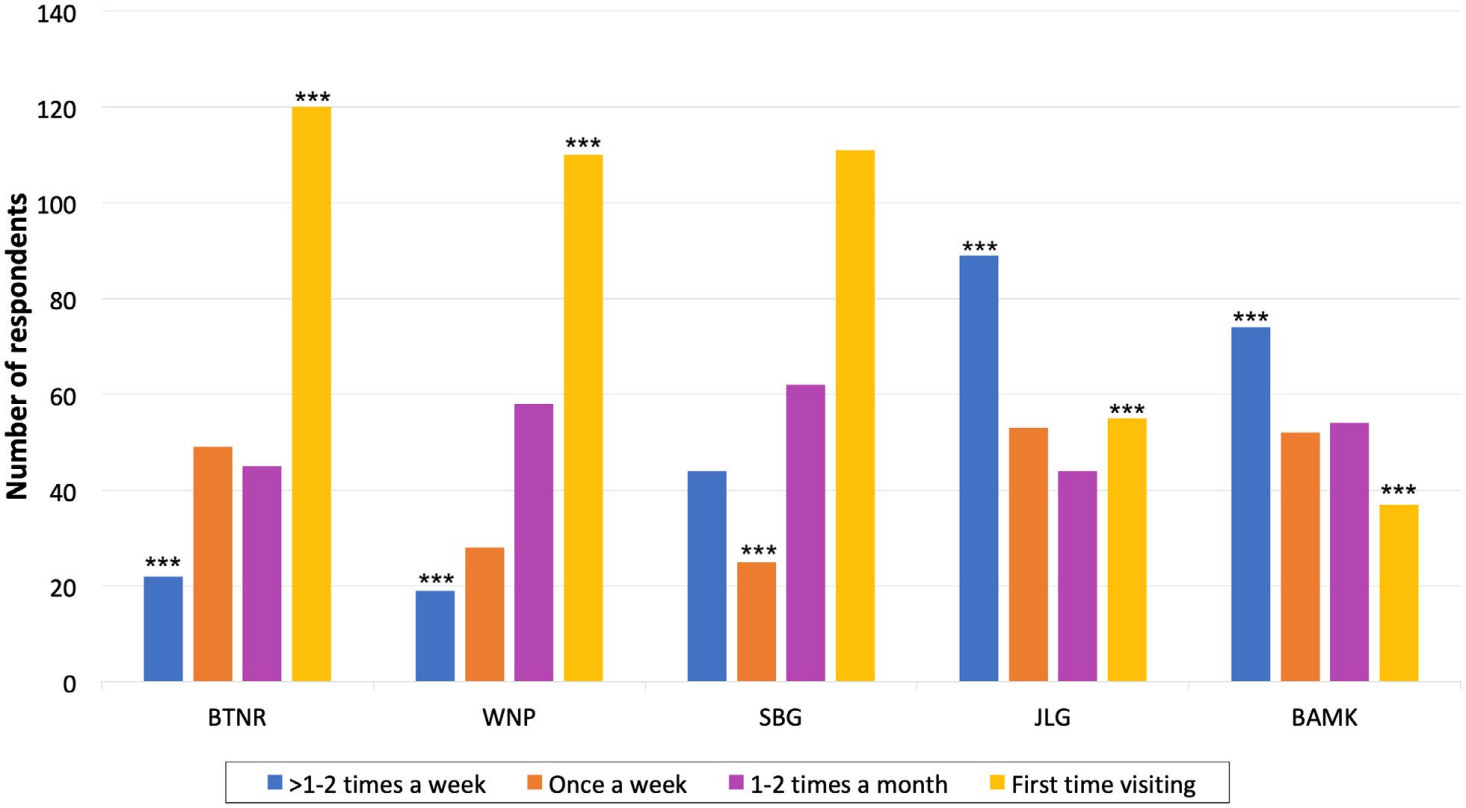
Self‐reported park visitor patterns during and post‐circuit breaker (*n* = 1,151). Frequency of visits to the parks differed across the five parks, ordered left to right from highest intensity of nature to most manicured (*χ* ^2^ = 165.18, *p* < 0.001). A chi‐square post‐hoc test was done to find out which frequencies contribute more towards this significant difference between the parks. *** indicates the chi‐square post‐hoc test significant differences at *p* < 0.001; **p < 0.01; *p < 0.05

37.6% of respondents were visiting that park for the first time since Circuit Breaker. First time visitors were skewed towards younger age groups, with 48.3% of all respondents aged 15–29 being first‐time visitors. Visitors to the five parks had different visit frequency and patterns (*χ* ^2^ = 165.18, *df* = 12, *p* < 0.001; Figure 4). The proportion of first‐time visitors was significantly higher for BTNR and WNP (51% for both), as compared to JLG and BAMK, which have a higher number of respondents who visit the park frequently once a week or more (Figure 4). Most respondents at BTNR had to travel further to visit the park as compared to those at JLG and BAMK where 45.6% and 51.1% of the visitors stayed within walking distance of the park.

Visitors were generally most likely to describe that they enjoyed the park for exercising, followed by appreciating the natural landscapes (Table 6). Spending time with others was the third most cited reason, with 49% choosing this option, and 32.3% enjoy watching wildlife in the park. The order of preference for first‐time visitors was different, with appreciation of the natural landscape coming as the top choice (70.7%) and exercise second (62.8%). All visitors primarily chose to visit parks for relaxation and leisure, but 28.8% of respondents responded that they visited parks as they were bored from staying at home. A number of respondents selected to provide their own responses, and among these, most of them stated that they went to the parks to tend to their allotment plot (allotment plot refers to a public gardening space that is rented by an individual).

Nearly all respondents answered positively with regard to their perception of their personal value of greenery since the COVID‐19 outbreak and seeing the public green spaces grow wilder during the Circuit Breaker (Table 6). Almost all park visitors were aware of measures taken for safe distancing in parks and expressed confidence in the management of safe distancing in parks during and after Circuit Breaker (Table 6). The preference for connecting with nature also appeared to apply to other green areas outside of parks, as 94.3% of those surveyed responded positively to how the roadsides have grown ‘wild’ due to reduced maintenance activities during the Circuit Breaker.

## 4 DISCUSSION

### 4.1 Effect of circuit breaker on interest and use of parks

The implementation of restrictions during the Circuit Breaker reduced online interest in parks, but the trend was reversed immediately after Circuit Breaker was lifted. In fact, all parks except one had higher search proportions than before the Circuit Breaker, with 12 out of 18 parks having a significant increase. The only park that did not show an increase in searches was the SBG, a UNESCO World Heritage site that is popular among tourists and well known for hosting large festivals and events. During the pre‐Circuit Breaker period from January to March 2020, SBG hosted 10 large events that drew thousands of people each time and people would tend to search for them via online channels. We speculate that this anomaly could be contributed to two main factors. First, a large proportion of SBG visitors comprised tourists during pre‐COVID times, which was greatly reduced due to travel restrictions (Elangovan, 2020). Second, large events and festivals had not yet resumed due to restrictions on the size of participants for events.

The spike in interest for parks in general after Circuit Breaker is further corroborated by similar trends shown via Google Mobility Data for parks. Right after the Circuit Breaker ended, visits to parks increased at a similar pace compared to other categories at the start, and a month later even experienced an increase in visitorship rates faster than retail and recreation when they were reopened. This is not surprising seeing that 45.4% of the park users have resumed their regular visit patterns to the parks before the pandemic and another 33.4% of visitors increased their use of the parks compared to before. This behavioural pattern of increased visitorship to parks was also observed in other parts of the world (Ma et al., 2021; Ugolini et al., 2020), and in some areas sustained visitorship too (Venter et al., 2021). This trend of park visitorship is likely to continue as almost all the respondents said that they will continue to visit parks in the new normal.

These results from google search and mobility data are in line with our predictions of a surge in interest in parks after a period of nature deprivation during the Circuit Breaker. The higher Google search rate also supports the notion that people who were previously not regular park visitors drove the surge in interest in parks. This hypothesis was confirmed by the survey results, where 63.3% of respondents said that they had visited a new park since the Circuit Breaker and 37.6% were visiting the park for the first time when they took the survey. This high rate of first‐time visitors was also found in similar studies during the COVID‐19 pandemic (Grima et al., 2020). Related searches for the parks included terms such as ‘hiking’ and ‘trail’, which emphasised the need for outdoor recreation (Kleinschroth & Kowarik, 2020).

### 4.2 Preference for type of UGS

The implementation and subsequent relaxation of mobility restrictions resulted in an increase in interest in parks, particularly in less manicured parks. Before the Circuit Breaker, Google searches for manicured parks were much higher than less manicured parks. There could be several reasons for this, such as, manicured parks having an array of shops and restaurants and having presence of open spaces for group activities. However, after the Circuit Breaker, interest in less manicured parks surpassed pre‐Circuit Breaker levels by 70.9%, and after the increase was on par with the interest for manicured parks, that only had only increased by 20.8%. The increased interest in less manicured parks was also supported by the disproportionate increase in the visitor counts in parks. Among the three parks, only the Nature Reserve (BTNR), a less manicured park, had the largest positive change in visitor counts compared to pre‐Circuit Breaker.

Many recent studies on the perception and use of UGS during the pandemic have emphasised the critical role of UGS in cities, citing its particular importance where access to other lifestyle activities is hindered (Kleinschroth & Kowarik, 2020; Soga et al., 2021; Yang et al., 2021). Our results suggest that this demand for UGS is differentiated by types of UGS, with a higher preference for recreation in a less manicured or wilder landscape in urban areas. This finding is comparable to the study by Grima et al. (2020) in Vermont, USA, where 70% of respondents claimed to have increased their visitation rates to natural spaces in urban areas and attributed a greater importance to them in the pandemic. Another similarity is that respondents ranked exercise and enjoying nature at the UGS above social activities, which can be attributed to the nature of the pandemic and restrictions imposed by the government. If people preferentially seek our less manicured parks, the implication is also that less manicured parks, compared to more manicured parks, are better able to meet the needs of Singapore residents during the pandemic. In other words, parks should not be viewed as homogeneous green spaces—there are different ‘shades of green’ that provide different levels of cultural ecosystem services as revealed by different sources of data in our study.

Interest in parks was also driven by two other key factors, namely, the size of the park and number of shops available. Several studies have reported positive correlations between park size and park use (Gu et al., 2020; Talal & Santelmann, 2021; Vierikko et al., 2020). Park users also showed a greater preference towards larger green spaces (Wang et al., 2015). Users preferred larger parks due to the increased perceived variety and opportunities to relax within the park (Corti et al., 1996), and thus also increases their motivations to visit (Halkos et al., 2021; Vierikko et al., 2020). In our study, larger parks provide more space (or distance in terms of trails) and amenities for exercising, which was the main activity that visitors used parks for. Commercial spaces that include food and beverage outlets (referred to as Shopping and Restaurants Places of Interests or SRPOIs) attract visitors to parks and open spaces (Chen et al., 2016). Apart from children's playgrounds and toilets, SRPOIs form key park facilities and are positively correlated with park user density (Chen et al., 2018). Our results showed that retail shops provide opportunities for social interactions, which were important for 49.4% of park visitors.

### 4.3 Inclination towards nature

We discuss our results in relation to people's connection to nature in this section. The overall increase in interest to visit parks, accompanied by an increase in less manicured parks, and the appreciation of natural landscape as the top reason for visiting parks among first‐time visitors converge as evidence that in Singapore, the COVID‐19 pandemic has heightened the need for residents to be in contact with nature. This is against the backdrop that local studies have shown that there was no extinction of experience of nature prior to the pandemic, and 76% of the population in a large cohort study visited parks monthly, and that life satisfaction is in fact, closely related to nature (Chang et al., 2020; Oh et al., 2020; Petrunoff et al., 2021). Therefore, the mobility restrictions, together with the stresses arising from COVID‐19, has increased the demand for contact with nature even in a city with high park visitorship.

Our results also point to an interesting phenomenon—there was a temporary deprivation of contact with nature during the Circuit Breaker, which when relieved, led to a surge in park visits. Even though parks remained open, government restrictions during the lockdowns reduced the opportunity for people to use parks as people stayed at home. These include closure of carparks at parks to discourage people from travelling beyond their immediate residential vicinity to visit parks. People were also strongly encouraged to only exercise in parks that are close to their homes and only individually at the height of Circuit Breaker. Increased confinement indoors during the Circuit Breaker led to reduced visit to parks. We posit that one pathway that could be driving this trend may be the ‘extinction of experience’ in nature (Louv, 2005; Soga & Gaston, 2016) brought about by the lockdown. Prolonged confinement and limited access to nature during the COVID‐19 pandemic may result in a temporary nature deprivation to urban dwellers, through reduced opportunity to access UGS, and bring about a decline in emotional and mental states (Nadkarni et al., 2017). The COVID‐19 lockdown in different parts of the world triggered mental and emotional stress as people refrained or were curbed from having social interactions as they were largely confined to their homes (Douglas et al., 2020). For example, studies reported an increase in stress and anxiety of students due to confinement (Giuntella et al., 2021; Husky et al., 2020; Zhang et al., 2020). The build‐up of stresses provoked behaviour and actions to try to alleviate them (Lechner et al., 2020), of which an outlet for relaxation was being in nature (Berdejo‐Espinola et al., 2021). We found this to be true for Singapore during the pandemic. Most of the survey respondents agreed that they visited parks to relax.

The rapid bounce back to experiencing nature after restrictions were lifted is also in line with the Health Belief Model (HBM) (Rosenstock, 1974). With strong evidence on the effects of confinement causing declining mental health during the COVID‐19 lockdowns, the model explains a sudden switch to pro‐nature lifestyles as people sought out opportunities for health and wellness benefits in UGS during the pandemic (Ramkissoon, 2020). The spike at BTNR was fuelled by a large proportion of first‐time visitors to the park (51% of survey respondents) who visited the parks to primarily enjoy the natural landscapes and not for exercise. Experiencing these green spaces allows for restoration of psychological health (Akpinar et al., 2016; Subiza‐Pérez et al., 2020) and the perceived animal diversity of the green space has been shown to improve emotional well‐being (Nghiem et al., 2021).

The pathway in which the COVID‐19 pandemic influences the cultural ecosystem services of UGS is important for future planning (Soga et al., 2021). Although the findings from this study suggest that these pathways describe reasons for the observations, the study has its limitations. The study would be able to draw more conclusive remarks on the effects of the extinction in nature experience, if there was a cross‐reference to the respondents' mental well‐being in the Circuit Breaker phases. In addition, surveys did not measure the state of orientation of the respondents to nature prior to the Circuit Breaker. Knowing their inclinations towards nature would provide a better understanding of how likely respondents would continue to want to experience nature even when opportunities are reduced (Colléony et al., 2020). The COVID‐19 pandemic happened suddenly, and it provided an unprecedented opportunity for us to study this unique global social phenomenon and study park usage patterns using a fresh baseline.

## 5 CONCLUSION

Prolonged home confinement through the implementation of travel restrictions during the height of the COVID‐19 pandemic was a stimulus that changed behaviour and preference of city dwellers towards parks and green spaces. COVID‐19 restrictions allowed the opportunity to understand the cultural ecosystem services provided by different types of UGS. The combination of multiple data sources—big data and physical surveys—have converged to conclude that the interest in and visits to parks became higher than it was before the pandemic, for relaxation and enjoyment of less manicured and natural landscapes. Looking beyond the pandemic in the new normal, it is important that urban nature and green spaces continue to be accessible to all. In Singapore, this further reinforces the important of the park planning target in Singapore to ensure that each and every person can access a park or green space within a 10‐min walk of their homes. The implications on park planning policy would then be to ensure that Singapore has a connected network of diversity of green spaces, including access to less manicured parks that people can easily access and experience nature.

## CONFLICT OF INTEREST

The authors work in a government body that oversees parks and green spaces in Singapore. They declare that they have no known competing financial interests or personal relationships that could have appeared to influence the work reported in this paper.

## AUTHORS' CONTRIBUTIONS

K.K.L.Y. and K.B.H.E. conceived the ideas, designed the methodology and led the writing for this manuscript; W.J.C. assisted K.K.L.Y. with collection of data; K.K.L.Y. and M.C.K.S. analysed the data; M.C.K.S., S.A., A.S., P.Y.T. and W.P.A. assisted with the drafting of the manuscript and reviewed the literature. All authors vetted the manuscript drafts, gave final approval for publication and agree to be accountable for the aspects of work they conducted.

## DATA SOURCES

The Google mobility data and Google search trends were obtained from Google sources that are made publicly accessible for use. The sources are cited in text and in the references.

## Supporting information

Supinfo

## Data Availability

The data for visitor counts (Figure 3) and visitor survey (Figure 4 and Table 6) are deposited in the Dryad Digital Repository https://doi.org/10.5061/dryad.nvx0k6dtz (Yap et al., 2022).
