# Supplementary material for: The influence of the COVID‐19 pandemic on the demand for different shades of green
Source: People Nat (Hoboken). 2022 Feb 14;4(2):505–18. doi: 10.1002/pan3.10304 (PMC9088662; doi:10.1002/pan3.10304)
Supplement: Supplementary file 1 — Supinfo [file PAN3-4-505-s001.pdf]

NATIONAL PARKS

Park visitation survey

5 mins estimated time to complete

Instructions

For each question, please tick the checkbox(s) that best represents your answer.

Note: This survey is entirely anonymous and participation is completely voluntary. If at any point you are no longer comfortable filling out the survey please inform the staff.

If you have any questions about the survey, feel free to ask the staff or contact the principal researcher at kathleen_yap@nparks.gov.sg (mailto:kathleen_yap@nparks.gov.sg).

---

1. Which park are you taking this survey at?

- ◯ Bishan-Ang Mo Kio Park
- ◯ Bukit Timah Nature Reserve
- ◯ Jurong Lake Gardens
- ◯ Singapore Botanic Gardens
- ◯ Windsor Nature Park

2. Which of the following age groups do you belong to?

- ◯ 15 – 19 years
- ◯ 20 – 29 years
- ◯ 30 – 39 years
- ◯ 40 – 49 years
- ◯ 50 – 59 years
- ◯ 60 – 69 years
- ◯ 70 years & above

3. What is your nationality status?

[illegible]

- Employment Pass Holder
- 'S' Pass Holder
- Dependent Pass Holder
- Student Pass Holder
- Work Permit Holder

4. **What mode of transportation do you usually use to travel to this park?**

- Walk/ Jog
- Cycle
- Public transport (includes private hire & taxi)
- Drive

5. **How much time does it take you to travel to this park to using the above-mentioned transportation mode?**

- Less than 10 mins
- 10 to 30 mins
- 30 mins to 1 hour
- More than 1 hour

6. **How often do you visit this park since the start of Circuit breaker (on average)?**

Circuit Breaker measures were first introduced in April 2020.

- More than 1-2 times a week
- Once a week
- 1-2 times a month
- First time visiting since April 2020

7. **How has the frequency of your visit to this park changed, comparing now and before the Circuit Breaker?**

- More frequently now than before
- Less frequently now than before
- No difference

8. **What do you enjoy doing at this park?**

Select all that apply

- [ ] Exercise (i.e. walking, running, hiking etc.)
- [ ] Appreciate the natural landscape in the park

- [ ] Others

If others, please specify.

9. Which of the following are reasons that motivate you to visit the parks during and after the Circuit Breaker?

- [ ] Relax / de-stress
- [ ] Bored at home
- [ ] Would have preferred going to the malls, but they were closed
- [ ] Others

The following statements are regarding how the Circuit Breaker has affected your park visit patterns. Do you agree with the following statements:

10. "I resided in Singapore before and during the COVID pandemic"

Select 'No' if you live overseas and returned to Singapore during the pandemic

✕ NO | ✓ YES

11. “Since April, I visited parks that I never visited before, or parks that I have not been to in the last 1 year”

✕ NO | ✓ YES

12. “I appreciate parks and the greenery in my neighbourhood now more than before the pandemic”

✕ NO | ✓ YES

13. "I am happy seeing our roadside and open spaces grow a little wilder"

✕ NO | ✓ YES

14. "I will continue to visit parks just as often or more even when things become normal again”

✕ NO | ✓ YES

15. “I feel that this park has become more crowded than before Circuit Breaker”

✕ NO | ✓ YES

16. “I am aware of some of the measures in parks to maintain public safety during the pandemic”

✕ NO | ✓ YES

17. "I feel that the measures in parks to ensure public safety during the pandemic is sufficient.

✕ NO | ✓ YES

SUBMIT

POWERED BY
